# Parametric and heat affected zone study on CO_2_ laser cutting of acrylic

**DOI:** 10.1016/j.mex.2023.102125

**Published:** 2023-03-11

**Authors:** Chockalingam Palanisamy, MNE Efzan, Chin Chee Wen

**Affiliations:** Faculty of Engineering and Technology, Multimedia University, Melaka, Malaysia

**Keywords:** CO_2_ laser cutting of Acrylic material, Co_2_ laser, Acrylic, Scanning speed, Current, Nozzle-work gap, Material removal rate

## Abstract

Laser cutting is a non-contact machining employed for producing small, intricate shapes. The acrylic materials are widely used in many applications. The parametric and heat affected zone study of acrylic materials by using CO_2_ laser machining is attempted in this research to evaluate the process variables, laser scanning speed, current, and nozzle-work material gap.

• Research result indicate that the higher the current and the higher the cutting speed, result in higher the material removal rate
• Other parameter such as current and nozzle, work material gap are also significant impact on the cutting process of the acrylic material.
• In addition, heat affect zone increase with laser scanning speed.

## Graphical abstract

**Figure:**
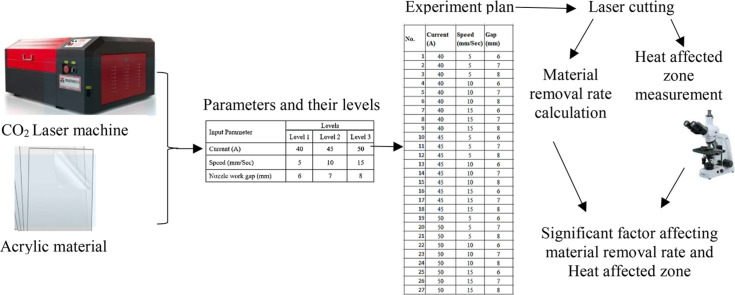

Specifications table

**Table:** 

Subject area:	Engineering
More specific subject area:	*Laser cutting*
Name of your method:	*Three factor three level design*
Name and reference of original method:	Shyha, I. (2013). An investigation into CO_2_ laser trimming of CFRP and GFRP composites. *Procedia Engineering, 63*, 931–937.
Resource availability:	EZ4040-M2 50 W Laser cutting machine With K40 Whisperer V0.57 control software https://www.scorchworks.com/K40whisperer/k40w_setup.html *Meiji Techno MT7000 Metallurgical microscope.*https://meijitechno.com/meiji_old/mt7000.htm

## Introduction

The use of laser machining processes is continuously increasing. CO_2_ laser machining is efficient in speed and accuracy. Laser machining can be effectively used on the metals, composites, acrylic and woods. Laser cutting can produce complex shapes at micrometre scale [1], [2], [3]. Laser machining is an ideal process for cutting materials effectively due to its high laser scanning speeds and flexibilities [4]. However, laser power may damage the materials thermally and affects their properties [5]. Many authors reported some experiment on CO_2_ laser cutting quality of polymer and composite material [6]. To understands the quality of laser cutting, some parameters like cutting width, heat affected zone and material removal rate are investigated. The laser cutting on polymers may damage the surfaces [7,8]. The essential parameters affecting polymeric laser cutting are, standoff distance, speed and beam power, surface roughness and heat affected zone [9,10]. In this research the effect of laser scanning speed, nozzle-work material gap and power on material removal rate (MRR), heat affected zone (HAZ) and cutting width of acrylic was investigated.

## Material and methods

Conventional machining has limitations because of size, shape, design and hardness of materials [11]. Laser cutting is used in all kind of materials such as metal, ceramic and plastics. The focused laser beam is guided and moved on the material to cut the required shape [12]. The ease of use of laser makes it possible to produce intricate shapes. Moreover, laser cutting gives a better finishing as compared to conventional cutting. A CO_2_ laser cutting machine with CNC capabilities (EZLaser 4040) was used to cut acrylic, using a hermetic sealed CO_2_ glass tube laser with 50 W power and a wavelength of 10.64µm. The machine was equipped with a stepper motor and a laser scanning speed ranging from 0 - 50 mm/s, using a long nozzle with 3 mm orifice diameter. The laser has a focusing diameter of 10 µm and focusing length ranging from 5 to 9 mm. The influencing process variables such as laser scanning speed, current, and nozzle-work material gap were investigated on the effect of cutting width, heat affected zone and material removal rate (MRR) [13]. The work material was set in flat position on machine table. The cutting was carried out on a 297 × 210 × 3 mm acrylic sheet for a length of 100 mm. The cutting was carried out by varying laser scanning speed, power and nozzle –work material gap. Its properties are good impact resistance, light weight. The performance of CO_2_ laser cutting was evaluated against MRR, cutting width and HAZ. The laser cutting parameters and their levels were given in the Table 1. The width and HAZ of cut was measured with an optical microscope. Power, nozzle-work material gap, and the laser scanning speed were varied in order to investigate the interaction behaviours. The CO_2_ laser is a multi-gas laser cutting machine. It mainly uses carbon dioxide, nitrogen, hydrogen and helium gas. The CO_2_ laser cutting is performed by combination of input parameters and significant results given in Table 2.

**Table 1:** Laser cutting process parameters and their levels.

Input Parameter	Levels		
	Level 1	Level 2	Level 3
Current (A)	40	45	50
Speed (mm/Sec)	5	10	15
Nozzle work gap (mm)	6	7	8

**Table 2:** Experiment plan and result.

No.	Current (A)	Speed (mm/*sec*)	Gap (mm)	Cut width (mm)	MRR (mm^3^/*sec*)	HAZ (mm)
1	40	5	6	1.32	19.8	0.4
2	40	5	7	0.75	11.25	0.36
3	40	5	8	0.75	11.25	0.39
4	40	10	6	0.64	19.2	0.39
5	40	10	7	0.59	17.7	0.42
6	40	10	8	0.59	17.7	0.35
7	40	15	6	0.61	27.45	0.41
8	40	15	7	0.51	22.95	0.4
9	40	15	8	0.55	24.75	0.43
10	45	5	6	1.08	16.2	0.39
11	45	5	7	0.85	12.75	0.4
12	45	5	8	0.75	11.25	0.37
13	45	10	6	0.84	25.2	0.39
14	45	10	7	0.64	19.2	0.38
15	45	10	8	0.58	17.4	0.38
16	45	15	6	0.52	23.4	0.41
17	45	15	7	0.58	26.1	0.39
18	45	15	8	0.57	25.65	0.42
19	50	5	6	1.32	19.8	0.43
20	50	5	7	0.82	12.3	0.43
21	50	5	8	0.95	14.25	0.4
22	50	10	6	0.75	22.5	0.43
23	50	10	7	0.63	18.9	0.37
24	50	10	8	0.67	20.1	0.39
25	50	15	6	0.56	25.2	0.38
26	50	15	7	0.57	25.65	0.44
27	50	15	8	0.61	27.45	0.44

## Results

**Fig. 1:**
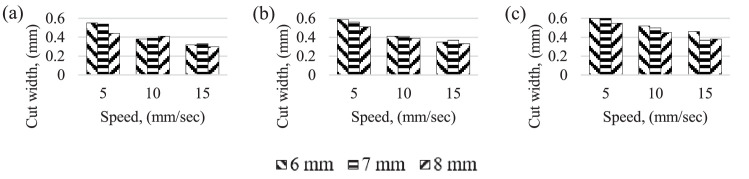
laser cut width for different speed and gap. (a) 40 A current (b) 45 A (c) 50 A current.

Fig. 1 show the laser cut width for different current. In general, for all current settings, laser scanning speed increases, cutting width decreases for all three current range tested and smaller the nozzle-work material gap wider the cutting width for the current setting except for 50 A current setting. The maximum cutting width of 1.32 mm achieved was for the 6 mm gap settings and smaller the width of the cutting was observed for the high laser scanning speed of 15 mm/*sec*. Finding also revealed that at high laser scanning speeds, narrow area of materials only melted.

**Fig. 2:**
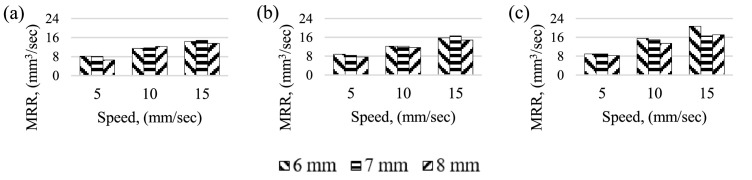
Material Removal Rate for different speed and gap. (a) Current, 40 A (b) Current, 45 A (c) Current, 50 A.

Fig. 2 illustrates material removal rate (MRR) as a function of laser scanning speed and nozzle-work material gap, under three different current settings. The MRR increased progressively with laser scanning speed for all current settings tested and regardless of the nozzle-work gap. Specifically, the MRR at a laser scanning speed of 15 mm/s was nearly twice that at a speed of 5 mm/s. At lower velocities, increasing the nozzle-work material gap leads to a decrease in MRR. The maximum MRR achieved was 20.7 mm^3^/*sec*, at a laser scanning speed of 15 mm/*sec*, a nozzle-work material gap of 6 mm, and a current setting of 50A. However, increasing the velocity and nozzle-work material gap significantly reduces the MRR for all three current settings.

**Fig. 3:**
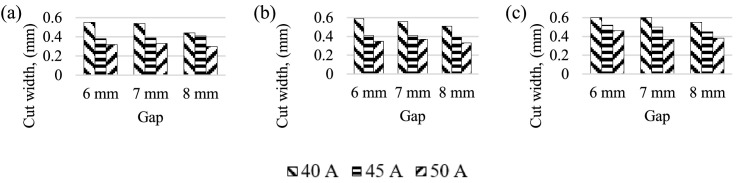
laser cut width for different current and gap. Laser scanning speed (mm/*sec*) (a) 5 (b) 10 (c) 15.

Fig. 3 shows that the width of cut decreases with an increase in the nozzle-work gap. Similarly, width of the cut decreases with increasing current. Lowest cutting width observed 0.3 mm for low laser scanning speed of 15 mm/*sec* and highest nozzle–work gap of 8 mm with 50A current. In addition, the decreasing trend of the cutting width could be explicated by the fact that a narrow width is due to high laser scanning speed laser beam not able to melt the work material. The laser beams contact time is more in lower laser scanning speed could result in wider cutting in the material [11]. Sequentially, more laser energy passing on to the material could results in a wider cut in the material [14].

**Fig. 4:**
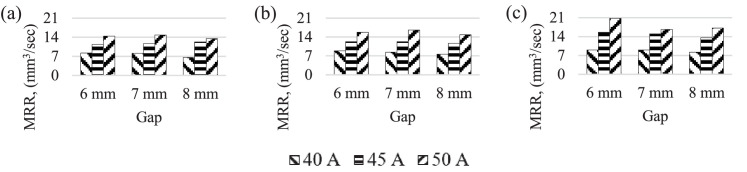
MRR for different current and gap. Laser scanning speed (mm/*sec*) (a) 5 (b) 10 (c) 15.

Fig. 4 shows the MRR for current and gap for different laser scanning speeds. MRR at higher laser scanning speeds and lower nozzle-work material gap. Furthermore, highest MRR obtained for 20.7 mm^3^/*sec* laser scanning speed with 6 mm nozzle-work material gap conditions at 50A current setting. This is due to the fact that the gap closer, laser energy increases, subsequently, the high laser scanning speed increased more materials removed. Lowest MRR, 6 mm^3^/*sec* observed for the slowest laser scanning speed, 5 mm^3^/*sec* at highest nozzle-work gap of 8 mm. This is due to fact that laser energy cannot get into the material at the highest laser scanning speed [15], [16].

**Fig. 5:**
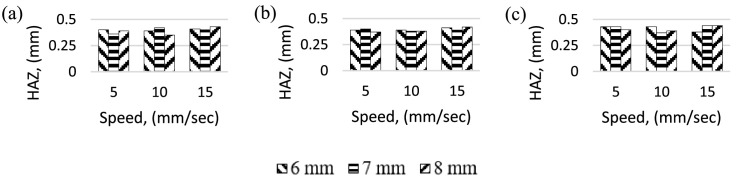
HAZ for different speed and gap. Current, 40 A (b) Current, 45 A (c) Current, 50 A.

The HAZ results of CO_2_ laser cutting acrylic material discussed here. The heat affected zone width was measured using an optical microscope (MEIJI Techno MT7000). Fig. 5 indicate that in general, it is evident that the HAZ decreases with the nozzle-work gap increases and HAZ increases with increase in laser scanning speed. Highest HAZ occurs at high laser scanning speed, high nozzle-work gap and high current setting. The current setting is directly proportional to laser power. According to Choudhury et al. (2010), HAZ is directly proportional to laser power [17], which is reflecting on this result. Similarly, lowest HAZ occurs at low laser scanning speed and low current settings.

**Fig. 6:**
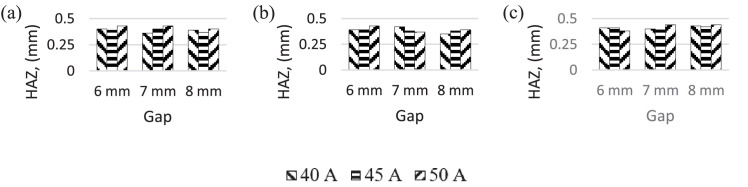
HAZ for different current and gap. Laser scanning speed (mm/*sec*) (a) 5 (b) 10 (c) 15.

In Fig. 6, it can be seen that the heat-affected zone generally decreased as the nozzle-work gap increased at low laser scanning speeds, but increased at high scanning speeds. Overall, the HAZ was not significantly influenced by the laser scanning speed. These results are consistent with those reported by Caiazzo et al. (2005) for plastics [18].

## Conclusion

A study of the influence of CO_2_ laser machining parameters on acrylic material was conducted, and based on the experimentations, the following conclusions were drawn. Higher laser scanning speeds results in lower cutting width. Similarly, a larger the nozzle-work gap results in a smaller cutting width. Higher current results in greater cutting in acrylic. Higher laser scanning speed result in higher material removal rate. However, a higher the nozzle-work material gap results in a lower MRR. As laser scanning speeds increase, HAZ increases. When the nozzle-work material gap increases HAZ decreases at low and medium laser scanning speeds, but increases at high laser scanning speeds.

## Declaration of Competing Interest

The authors declare no conflict of interest.

## Declaration of Competing Interest

The authors declare that they have no known competing financial interests or personal relationships that could have appeared to influence the work reported in this paper.

## Data Availability

No data was used for the research described in the article.
